# A Soft Coral-Derived Compound, 11-*epi*-Sinulariolide Acetate Suppresses Inflammatory Response and Bone Destruction in Adjuvant-Induced Arthritis

**DOI:** 10.1371/journal.pone.0062926

**Published:** 2013-05-13

**Authors:** Yen-You Lin, Yen-Hsuan Jean, Hsin-Pai Lee, Wu-Fu Chen, Yu-Min Sun, Jui-Hsin Su, Yi Lu, Shi-Ying Huang, Han-Chun Hung, Ping-Jyun Sung, Jyh-Horng Sheu, Zhi-Hong Wen

**Affiliations:** 1 Department of Marine Biotechnology and Resources, Asia-Pacific Ocean Research Center, National Sun Yat-Sen University, Kaohsiung, Taiwan, Republic of China; 2 Section of Orthopedic Surgery, Pingtung Christian Hospital, Pingtung, Taiwan, Republic of China; 3 Department of Neurosurgery, Chang Gung Memorial Hospital-Kaohsiung Medical Center, Chang Gung University College of Medicine, Kaohsiung, Taiwan, Republic of China; 4 Doctoral Degree Program in Marine Biotechnology, National Sun Yat-Sen University, Kaohsiung, Taiwan, Republic of China; 5 Graduate Institute of Marine Biotechnology, National Dong Hwa University, Pingtung, Taiwan, Republic of China; 6 Taiwan Coral Research Center, National Museum of Marine Biology and Aquarium, Pingtung, Taiwan, Republic of China; Southern Medical University, China

## Abstract

In recent years, a significant number of metabolites with potent anti-inflammatory properties have been discovered from marine organisms, and several of these compounds are now under clinical trials. In the present study, we isolated 11-*epi*-sinulariolide acetate (Ya-s11), a cembrane-type compound with anti-inflammatory effects, from the Formosa soft coral *Sinularia querciformis*. Preliminary screening revealed that Ya-s11 significantly inhibited the expression of the proinflammatory proteins induced nitric oxide synthase and cyclooxygenase-2 in lipopolysaccharide-stimulated murine macrophages. We also examined the therapeutic effects of Ya-s11 on adjuvant-induced arthritis (AIA) in female Lewis rats, which demonstrate features similar to human rheumatoid arthritis (RA). Animal experiments revealed that Ya-s11 (subcutaneously 9 mg/kg once every 2 days from day 7 to day 28 postimmunization) significantly inhibited AIA characteristics. Moreover, Ya-s11 also attenuated protein expression of cathepsin K, matrix metalloproteinases-9 (MMP-9), tartrate-resistant acid phosphatase (TRAP), and tumor necrosis factor-α (TNF-α) in ankle tissues of AIA-rats. Based on its attenuation of the expression of proinflammatory proteins and disease progression in AIA rats, the marine-derived compound Ya-s11 may serve as a useful therapeutic agent for the treatment of RA.

## Introduction

Rheumatoid arthritis (RA) is an autoimmune and chronic erosive inflammatory disease characterized by chronic edema and inflammation of the synovial tissue that lines joints. RA affects approximately 1% of the adult population worldwide, and RA patients present an average of 43% of maximum possible joint destruction after 20 years of suffering from the disease [1]–[3]. As RA progresses, the lining of the joints degenerates, leading to articular destruction and decreased joint mobility with radiological evidence of erosive damage and significantly impacting quality of life within 2 years of disease onset [4], [5]. Over the past 2 decades, more effective therapeutic strategies for RA including synthetic modifying antirheumatic drugs and/or biologic agents have been developed, but they carry potential risks. Additionally, nonsteroidal medications and corticosteroids are required as adjunctive therapy [6]. Therefore, new drug development for RA remains essential.

Prior studies have indicated that inflammatory processes are critical to the development of RA [2], [7], [8]. Synovial inflammation causes hyperplasia of the synovial tissue, with clusters of large numbers of infiltrating cells, and a tumor-like structure called pannus invades the joint lining and destroys local articular structure [9], [10]. Although the precise etiology of RA remains unclear, macrophages, neutrophils, lymphocytes, and synovial fibroblasts in hyperplastic synovial tissue have been identified as major participants in the initiation and development of RA [11], [12]. These infiltrating cells can release proinflammatory cytokines such as tumor necrosis factor alpha (TNF-α) that mediate synovial inflammation and joint destruction [8], [13]. In addition, proinflammatory cytokines activate synovial fibroblasts and chondrocytes and lead to upregulation of osteoclast-related proteins including cathepsin K, matrix metalloproteinase-9 (MMP-9), and tartrate-resistant acid phosphatase (TRAP), which also participate in inflammatory arthritis resulting in joint destruction [14].

Lipopolysaccharide (LPS)-challenged murine macrophages are widely used for *in vitro* anti-inflammatory screening of terrestrial- and marine-derived natural compounds [15]–[17]. LPS activates the nuclear factor-κB pathway to massively upregulate the proinflammatory proteins inducible nitric oxide synthase (iNOS) and cyclooxygenase-2 (COX-2) in murine macrophages [18]. Several studies have indicated that macrophages play a critical role in RA by expressing pro-inflammatory proteins in inflamed synovial tissue and at the cartilage-pannus junction [11], [19]. They also release cytokines and chemokines that mediate inflammation and form a complex cytokine network with neutrophils, lymphocytes, and synovial fibroblasts in RA [13]. Macrophages are also important for their capacity to differentiate into osteoclasts, multinucleated giant cells, or mononuclear precursor cells. A number of previous studies have employed macrophage-related cell lines to investigate RA-related mechanisms of action and identify the anti-arthritic activity of compounds [8], [20]–[22]. Adjuvant-induced arthritis (AIA) is also widely used as an animal model for the study of clinical RA. Several potential anti-RA agents have been discovered from this model [1], [8], [23].

In recent years, a significant number of metabolites with potent bioactive properties have been discovered in marine organisms, and several of these compounds are now under clinical trials [24], [25]. In this study, we aimed to examine an anti-inflammatory cembrane-type compound for its ability to suppress RA progression. We isolated 11-*epi*-sinulariolide acetate (Ya-s11), a known cembrane-type compound, from the soft coral *Sinularia querciformis*. We found that Ya-s11 inhibited expression of the pro-inflammatory proteins iNOS and COX-2 in LPS-induced murine RAW264.7 macrophages. We also evaluated the anti-inflammatory and anti-rheumatic effects of Ya-s11 in AIA rats.

## Materials and Methods

### Preparation of 11-*epi*-sinulariolide acetate

**Figure 1:**
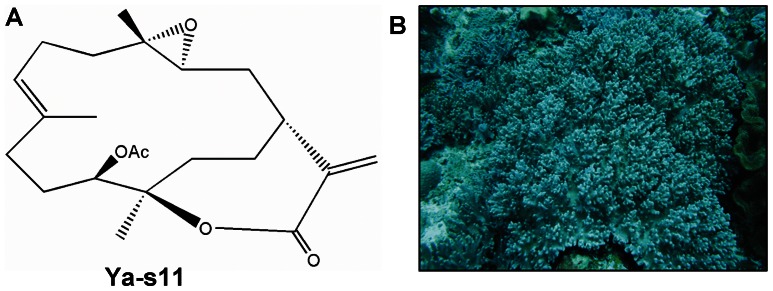
Chemical structure and source of 11-*epi*-sinulariolide acetate (Ya-s11). (A) The chemical structure of 11-*epi*-sinulariolide acetate. Molecular formula, C_22_H_32_O_5_, Molecular weight, 376. (B) The soft coral sample, *Sinularia querciformis*, was collected from Dongsha Island.

In the present study, 11-*epi*-sinulariolide acetate (Ya-s11, structure shown in Fig. 1) was isolated and purified from non-protected soft coral, *S. querciformis*, collected from the Dongsha Islands, Taiwan in 2008. We state that no specific permissions were required for sample collection in Dongsha Islands. Moreover, we also state that the area was not privately owned or protected. The method of 11-*epi*-sinulariolide acetate extraction was modified from that of Lu et al. [26], [27]. The soft coral specimens were minced and exhaustively extracted with 95% ethanol (8 liters). The crude extract was concentrated to an aqueous suspension and further partitioned between *n*-hexanes, ethyl acetate, and H_2_O. The *n*-hexaneethyl acetate layer was separated over normal phase silica gel by column chromatography and eluted with *n*-hexane, ethyl acetate, acetone, and methanol to yield 29 fractions. Fraction 11 was further eluted with *n*-hexanes and acetone (1∶1) over normal phase silica gel to generate 5 subfractions. Subfraction 4 was further separated by reverse-phase RP18 gel elution with H_2_O and acetonitrile (2∶1) by column chromatography to yield Ya-s11. The structure of Ya-s11 was confirmed by nuclear magnetic resonance spectroscopy (NMR) [28]. The purity (>98%) of Ya-s11 was determined and verified by 1H-NMR and 13C-NMR spectra (Varian Mercury Plus 400 FT-NMR at 400 MHz, Varian, CA, U SA).

### *In vitro*, anti-inflammatory activity assay

The anti-inflammatory activity assay was modified from Jean et al. (2008) and Chen et al. (2011) [15], [29]. RAW 264.7 murine macrophages were supplied by the American Type Culture Collection (ATCC, No. TIB-71) and grown in DMEM (including 10% heat-inactivated fetal bovine serum, 2 mM glutamine, 1 mM pyruvate, 4.5 g/l glucose, 50 mg/ml streptomycin, and 100 U/ml penicillin G) at 37 °C in a humidified 5% CO_2_: 95% air incubator. Inflammatory response was induced by incubating RAW264.7 cells for 18 h in DMEM containing only LPS (0.01 µg/ml) without the other added compounds. For the anti-inflammatory activity assay, Ya-s11 (1, 10, 25, or 50 µM) was added to the medium 10 min before LPS treatment of RAW264.7 murine macrophages. Cell pellets were collected by washing with ice-cold phosphate-buffered saline (PBS) and then lysed in 4 °C lysis buffer (50 mM Tris, pH 7.5, 150 mM NaCl, 1% Triton X-100, 100 µg/ml phenylmethylsulfonyl fluoride, 1 µg/ml aprotinin). After centrifuging at 14,000 rpm for 30 min at 4°C, the supernatant was obtained from the pellet and prepared for western blot analysis of pro-inflammatory proteins, iNOS and COX-2. Protein concentrations were quantified using the DC protein assay kit (Bio-Rad, Hercules, CA, USA) modified from the assay of Lowry et al. (1951) [30]. Western blotting was performed as described in our previous study [15]. An equal volume of sample buffer (2% sodium dodecyl sulfate (SDS), 10% glycerol, 0.1% bromophenol blue, 2% 2-mercaptoethanol, and 50 mM Tris–HCl, pH 7.2) was added to the supernatant and then loaded into a tricine SDS-polyacrylamide (7% or 10%) gel for electrophoresis at 120 V for 120 min. The proteins were transferred to a polyvinylidene difluoride membrane (PVDF; 0.45- µM pore size, Immobilon-P, Millipore, Bedford, MA, USA) at 135 mA overnight at 4 °C in transfer buffer (50 mM Tris–HCl, 380 mM glycine, 1% SDS, and 20% methanol). The membrane was blocked for 40 min at room temperature with 5% non-fat dry milk in Tris-buffered saline with Tween-20 (TTBS; 0.1% Tween 20, 20 mM Tris–HCl, 137 mM NaCl, pH 7.4) and then incubated overnight at 4 °C with primary antibodies directed against iNOS (1∶2,000 dilution; BD Pharmingen, San Diego, CA, USA; catalog no. 6103322; polyclonal antibody), COX-2 (1∶2,000, Cayman Chemical, Ann Arbor, MA, USA; catalog no. 160106; polyclonal antibody), and β-actin (1∶2,000, Sigma, St. Louis, MO, USA; catalog no. A5316-2ML; monoclonal antibody). The iNOS, COX-2, and β-actin antibodies recognized bands at ∼135, ∼72, and 45 kDa, respectively. The immunoreactive bands were visualized by enhanced chemiluminescence (Millipore, Billerica, MA, USA) and the Biochemi Imaging System, and relative densitometric quantification was performed using LabWorks 6.2 software (UVP, Upland, CA, USA).

### *In vivo* study

#### Animals

Female Lewis rats (180–220 g) were used for the experiments and obtained from National Laboratory Animal Center, Taiwan. The rats were maintained in plexiglass cages in a temperature-controlled (24±1 °C) room on a 12-h light/dark cycle and given free access to food and water. Each rat was used only once during the experiment. All drug injections were performed under isoflurane anesthesia. The use of animals accorded to the Guiding Principles in the Care and Use of Animals of the American Physiology Society and was approved by the institutional animal care and use committee of National Sun Yat-sen university. Every effort was made to minimize the number of animals used and their suffering.

### Adjuvant induce arthritis (AIA) and compound treatment

The method of generating AIA rats was modified from that of Sano et al. (1992) and Turull and Queralt (2000) [31], [32]. Heat-killed and lyophilized *Mycobacterium butyricum* was suspended in incomplete Freund's adjuvant 10 mg/ml (Sigma, St. Louis, MO, USA) on ice. Rats were immunized with an adjuvant injection of 10 mg/ml *M. butyricum* in incomplete Freund's adjuvant. On day 0, rats were injected intradermally at the base of the tail with 0.1 ml of adjuvant, and the development of arthritis was monitored from day 0 to day 28. Lewis rats were randomly divided into 5 groups: AIA (*n* = 7), AIA plus Ya-s11 (3 mg/kg) (*n* = 6), AIA plus Ya-s11 (9 mg/kg) (*n = *6), naïve (*n = *6), and Ya-s11 (9 mg/kg) treatment alone (*n = *6). In the AIA plus Ya-s11 groups, rats received 10 times of Ya-s11 at 2-day intervals between days 7 and 28. All rats underwent measurement of hindpaw edema and clinical evaluation at day 0 and before every Ya-s11 injection between days 7 and 28, and then rats were sacrificed on day 28 for histopathological analysis and immunochemical staining. The extent of edema in the foot and hindpaw was measured from day 0 (baseline) to day 28 after AIA using a plethysmometer (Paw Volume Meter, Singa, Taiwan). Rats were evaluated for arthritic processes every 2 days using a macroscopic scoring system, with score 0 = no signs of arthritis, 1 = swelling and/or redness of the paw or 1 digit, 2 = two tow joints involved, 3 = more than two joints involved, 4 = severe arthritis of the entire paw and all digits [9], [33], [34]. The macroscopic score for each rat was calculated by adding the scores of each individual paw [9], [34].

### Histopathological examination and immunohistochemical staining

Rats were sacrificed by perfusion with ice-cold PBS and 4% paraformaldehyde on day 28 after immunization, and ankle joints were removed and fixed in 10% neutral formalin for 4 days. The ankle joints were decalcified with 12.5% ethylenediaminetetraacetic acid (EDTA) in 10% neutral formalin for 2 weeks and then sectioned on the sagittal plane through the center of samples. The specimens were dehydrate in a graded series of alcohol (Tissue-Tek, Sakura Finetek Japan Co., Ltd, Japan), embedded with paraffin, and cut into 2- µm sections (Microm HM550, Microm, Waldorf, Germany for hematoxylin and eosin (H&E) and immunohistochemical staining. General and pathological changes in morphology were assessed by microscopic examination using an upright microscope for higher magnification (DM 6000B, Leica Inc., Germany) and a stereomicroscope for lower magnification (APO Z16, Leica Inc., Singapore) with a microscope digital image output system (SPOT idea 5.0 Mp Color Digital Camera, Diagnostic Instruments Inc., Sterling Height, MI., USA). To quantitatively evaluate joint destruction in the ankle, the degree of morphologic changes in each group was scored on photomicrographs of tissue sections, with score 0 = no damage, 1 = edema, 2 = presence of inflammatory cells, and 3 = cartilage and bone damage (Cuzzocrea et al., 2005). Infiltrating cells were further quantified according to the histopathological features of neutrophils, lymphocytes, macrophages, and synovial fibroblasts [35]–[37].

Ankle joint specimens were processed for immunohistochemical analysis as described in previous studies [38]–[40]. Paraffin-embedded ankle joint sections were placed on slides, deparaffinized with xylene, and dehydrated in a graded series of alcohol, after which endogenous peroxidase activity was quenched by 30-min incubation in 0.3% hydrogen peroxide. The antigen was retrieved by enzymatic digestion with proteinase K (20 mM; Sigma) in PBS for 20 min. After washing in ice-cold PBS, slides were incubated at 4 °C for 48 h with anti-cathepsin K (1∶100, Abcam, Cambridge, MA, USA; catalog no. ab19027; polyclonal antibody), anti-MMP9 (1∶100, Abcam; catalog no. ab76003; monoclonal antibody.), anti-TNF-α (1∶100, AnaSpec, Fremont, CA, USA; catalog no. 55383; polyclonal antibody), or anti-TRAP (1∶100, Santa Cruz, Delaware Avnue Santa Cruz, CA, USA, catalog no sc-28204; polyclonal antibody) in a humidified chamber. The sections were incubated for 90 min with biotinylated anti-rabbit IgG (Vector Laboratories, Burlingame, CA, USA) diluted 1∶200 in 1% bovine serum albumin (BSA) in PBS. Sections were then immunohistochemically labeled with the avidin-biotin complex technique using an ABC kit (Vectastain ABC kit; Vector Laboratories). Finally, the sections were treated with 3,3′-diaminobenzidine tetrahydrochloride (DAB; Peroxidase substrate kit, Vector Laboratories) for 1–5 min. All slides for immunohistochemistry were analyzed under a light microscope (DM 600B, Leica Inc. Wetzlar, Germany) with microscope digital image output system.

### Statistical analysis

All data are presented as mean ± SEM. For the immunoreactivity data, each test band is shown as the integrated optical density (IOD) computed with respect to the average optical density of the corresponding control (LPS-only treatment) band. The data were analyzed using 1-way analysis of variance (ANOVA) followed by Student-Newman-Keuls *post hoc* test (SigmaStat 3.5 for Windows). Differences resulting in *P* values less than 0.05 were considered significant.

## Results

### The inhibitory effects of Ya-s11 on iNOS and COX-2 protein expression

**Figure 2:**
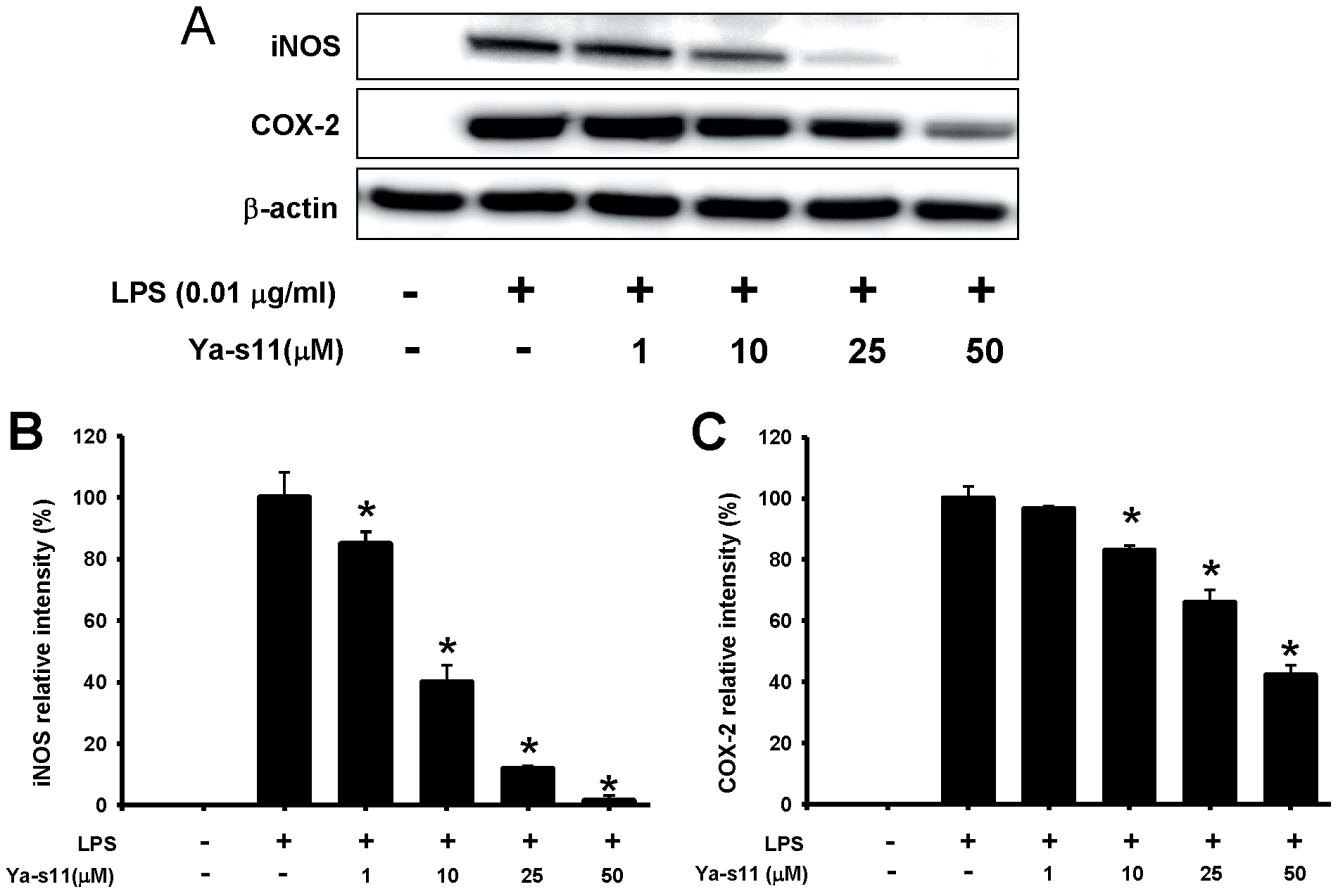
Effect of Ya-s11 on pro-inflammatory iNOS and COX-2 protein expression in LPS-stimulated macrophage cells. (A) immunoreactive bands corresponding to iNOS, COX-2, and β-actin protein from RAW 264.7 cells; (B) relative density of iNOS immunoreactive bands; (C) relative density of COX-2 immunoreactive bands. The relative intensity of the LPS group was set to 100%. Band intensities were quantified by densitometry and are indicated as the percent change relative to that of the LPS group. Western blotting with β-actin was performed to verify that equivalent amounts of protein were loaded in each lane. Ya-s11 significantly inhibited LPS-induced iNOS and COX-2 protein expression in murine Raw 264.7 macrophage cells. The experiment was repeated 4 times. *P<0.05, significantly different from the LPS-induced group.

The dose inhibition of 11-*epi*-sinulariolide acetate (Ya-s11) on LPS-induced pro-inflammatory 130-kDa iNOS and 71-kDa COX-2 protein expression is shown in Figure 2. At 1, 10, 25, and 50 µM doses of Ya-s11, iNOS levels were significantly reduced to 84.89±8.23%, 39.89±5.64%, 11.8±1.03%, and 1.4±1.74% relative protein expression, respectively, compared to the cells treated only with LPS (Fig. 2B). At 10, 25, and 50 µM concentrations, Ya-s11 significantly reduced COX-2 levels to 82.89±1.63%, 65.93±4.22%, 52.63±4.76%, and 42.13±3.25% relative protein expression compared to the cells treated only with LPS (Fig. 2C). Levels of β-actin protein (internal control) demonstrated no significant difference between concentrations of 1, 10, 25, and 50 µM Ya-s11 or compared with LPS only. Thus, Ya-s11 demonstrated concentration-dependent inhibition of LPS-induced iNOS and COX-2 protein expression in RAW 264.7 murine macrophages. Additionally, at the experimental concentrations, Ya-s11 did not induce cytotoxicity as determined through trypan blue staining and Annexin-V/PI double-staining assay (Figure S1).

### Effect of Ya-s11 on the clinical features of AIA

**Figure 3:**
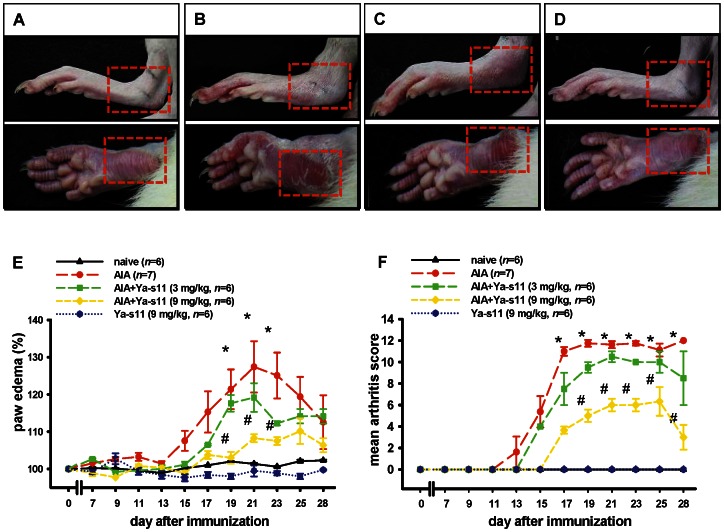
Effect of Ya-s11 on AIA rats. Typical representative macroscopic photographs of ankle and paw from the (A) naïve, (B) AIA, (C) AIA+Ya−s11 (3 mg/kg), and (D) AIA+Ya−s11 (9 mg/kg) groups. The AIA and AIA+Ya−s11 (3 mg/kg) groups displayed significant the edema on ankle joints and erythema on hindpaws (red square) compared to the naïve group (C). The AIA+Ya−s11 (9 mg/kg) group demonstrated apparent reduction of AIA-induced edema and erythema (D). Quantitative analysis of the effect of Ya-s11 at doses of 3 or 9 mg/kg on AIA-induced paw edema (E). Baseline values for the paw volume of each rat were set to 100%, and changes in edema level were calculated as a percentage increase from the control (pre-drug) volume. Ya-s11 demonstrated dose-dependent inhibition of AIA-induced paw edema in rats. Clinical evaluation of the effect of Ya-s11 on AIA-induced clinical signs in rats (F). Ya-s11 demonstrated a dose-dependent effect on AIA-induced clinical signs. Ya-s11 (3 or 9 mg/kg) was subcutaneously injected every 2 days between day 7 and day 28. Values reflect the mean ± SEM for each group. *P<0.05 compared with the naïve group. ^#^ *P*<0.05 compared with the AIA group.

AIA developed rapidly in rats immunized with heat-killed *M. butyricum*, and Figure 3 show the square of typical representative macroscopic photographs. Both AIA and AIA plus Ya-s11 (3 mg/kg) groups demonstrated edema and erythema on the ankle joints and hindpaws (Fig. 3B and C). Ya-s11 (9 mg/kg) significantly attenuated the AIA-induced edema and erythema of the hindpaw (Fig. 3D). Figure 3E illustrates the time-dependent increase of paw edema in immunized rats. Paw edema significantly increased to approximately 121.4–127.4% of baseline values from day 19 to day 23 in the AIA group, and the AIA plus Ya-s11 (9 mg/kg) group demonstrated a dose-dependent inhibition of paw edema compared with the AIA-only group. Quantitative analysis using a macroscopic scoring system (Fig. 3F) revealed a significant reduction in the AIA-induced upregulation of arthritis score in the AIA plus Ya-s11 (9 mg/kg) group. The AIA plus Ya-s11 (3 mg/kg) group demonstrated a slight but not significant attenuation in arthritis score (Fig. 3F). No change in paw edema or arthritis score was observed in the group treated with Ya-s11 (9 mg/kg) (Fig. 3E-F).

### The effect of Ya-s11 on histological features of AIA in the ankle joint

**Figure 4:**
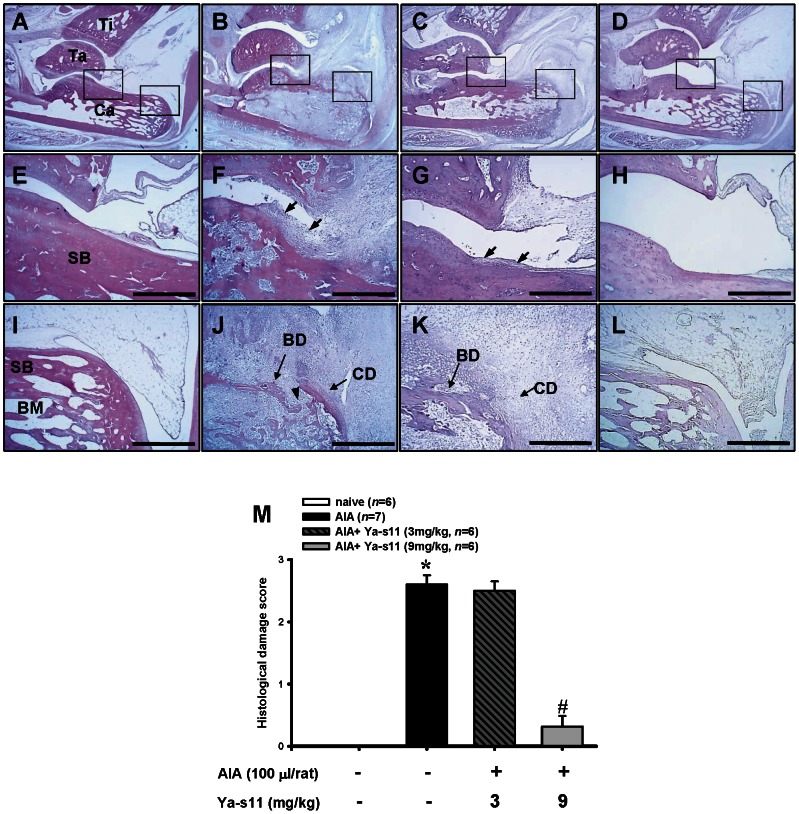
Histopathological assessments of the effect of Ya-s11 on the AIA rat ankle joint. Representative sections of ankle joint from the (A, E, I) naïve, (B, F, J) AIA, (C, G, K) AIA+Ya-s11 (3 mg/kg), and (D, H, L) AIA+Ya-s11 (9 mg/kg) groups stained with H&E. Normal joint structure showing calcaneus-talus articulation with the distal tibia and normal synovial tissue was observed in the naïve group (A). Marked joint destruction with bone damage, synovial tissue hyperplasia, and increased relative size of the marrow cavity of the subchondral bone marrow was observed in the AIA group (B). A higher-magnification view of AIA group shows pannus formation (arrow) and bone erosion over the rim of articular bone (F), and synovial tissue cells infiltration into subchondral bone marrow through the erosive orifice (arrowhead) into the calcaneus (J). The AIA+Ya-s11 (3 mg/kg) group demonstrated marked joint destruction with bone damage and synovial tissue hyperplasia (C). A higher-magnification view of AIA+Ya-s11 (3 mg/kg) group shows less severe pannus formation (arrow) and cartilage and bone destruction and resorption on the calcaneus compared with the AIA group (G). In the AIA+Ya-s11 (9 mg/kg) group, no morphological change in the ankle joint was apparent (H, L). The representative histopathological scores (M) of each group were analyzed to assess the degree of morphological changes, and the AIA+Ya-s11 (9 mg/kg) group demonstrated a significant decrease in the degree of arthritis. SB, subchondral bone marrow; Ca, calcaneus; Ta, talus; Ti, tibia; CD, cartilage destruction; BD, bone destruction; E–L, scale bar = 500 µm. *P<0.05 compared with the naïve group. ^#^ *P*<0.05 compared with the AIA group.

Rats were sacrificed on day 28 after immunization, and paraffin sections of ankle joints were subjected to H&E staining for histopathological analysis. Representative photographs of ankle joint sections are shown from control (Fig. 4A, E, I), AIA (Fig. 4B, F, J), AIA plus Ya-s11 (3 mg/kg) (Fig. 4C, G, K) and AIA Plus Ya-s11 (9 mg/kg) rats (Fig. 4D, H, L), respectively. Similar to previous studies [4], [41], in the AIA group, synovial tissue demonstrated synovial hyperplasia, cartilage and bone erosion (Fig. 4I), and moderate to severe infiltration of immune cells into subchondral bone marrow (Fig. 4J). The AIA plus Ya-s11 (3 mg/kg) group did not show attenuation of the synovial hyperplasia or cartilage and bone erosion, but moderated bone resorption in bone marrow (Fig. 4G, K). By contrast, the AIA plus Ya-s11 (9 mg/kg) group demonstrated significantly inhibited AIA-induced joint destruction and synovial hyperplasia, cartilage and bone erosion, pannus formation, and bone resorption (Fig. 4H, J). Histological damage scores were significantly higher in the AIA group compared to the naïve group. Those of the AIA plus Ya-s11 (9 mg/kg) group were significantly lower than in the AIA and AIA plus Ya-s11 (3 mg/kg) groups. No significant differences were observed between the AIA and AIA plus Ya-s11 (3 mg/kg) groups (Fig. 4M).

### Effect of Ya-s11 on infiltrating cells in synovial tissue

**Figure 5:**
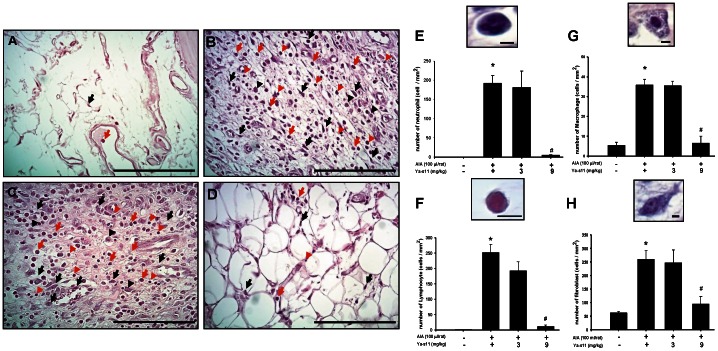
Effect of Ya-s11 on cell infiltration to the synovial tissue in AIA rats. Representative photographs of H&E-stained synovial tissue from the (A) naïve, (B) AIA, (C) AIA+Ya-s11 (3 mg/kg), and (D) AIA+Ya-s11 (9 mg/kg) groups. The synovial tissue of the naïve group demonstrated synovial fibroblasts with few immune cells (arrow) (A). Upregulation of neutrophils (red arrow), lymphocytes (red head arrow), macrophages (black head arrow), and synovial fibroblasts (black arrow) was observed in synovial tissue from the AIA group (B) and AIA+Ya-s11 (3 mg/kg) group (C). Ya-s11 (9 mg/kg) appeared to attenuate AIA-induced upregulation of immune cells. The numbers of neutrophils (E), lymphocytes (F), macrophages (G), and synovial fibroblasts (H) were analyzed in synovial tissue from each group. Higher-magnification views of synovial tissue from the AIA group shows histopathological features of infiltrating cells (E-H). Cell numbers significantly increased between the naïve group and AIA group and were significantly decreased in the AIA+Ya-s11 (9 mg/kg) group compared with the AIA group. ^*^ *P*<0.05 compared with the naïve group. ^#^ *P*<0.05 compared with the AIA group. A–D, scale bar = 100 µm; E–H, scale bar = 5 µm.

Pannus formation is accompanied by the infiltration of inflammatory cells, including lymphocytes, monocytes/macrophages, neutrophils, and synovial fibroblasts, into synovial tissue [2]. Representative photographs show synovial tissue stained with H&E from the naïve group (Fig. 5A), AIA group (Fig. 5B), AIA plus Ya-s11 (3 mg/kg) group (Fig. 5C), and AIA plus Ya-s11 (9 mg/kg) group (Fig. 5D). The synovial tissue of the naïve group demonstrated synovial fibroblasts with few immune cells (Fig. 5A), and marked upregulation of infiltrating cells was apparent in synovial tissue from the AIA group (Fig. 5B). Ya-s11 (9 mg/kg) significantly inhibited AIA-induced upregulation of infiltrating cells in synovial tissue (Fig. 5D). Neutrophils, lymphocytes, macrophages, and synovial fibroblasts all significantly increased between the naïve and AIA groups (Fig. 5E–R), and the AIA plus Ya-s11 (9 mg/kg) group demonstrated a significant decrease in the number of neutrophils, lymphocytes, and macrophages as well as inhibition of synovial fibroblast proliferation. No significant change was found in the AIA plus Ya-s11 (3 mg/kg) group compared with the AIA group.

### Effect of Ya-s11 on cathepsin K, MMP-9, TRAP, and TNF-α in AIA

**Figure 6:**
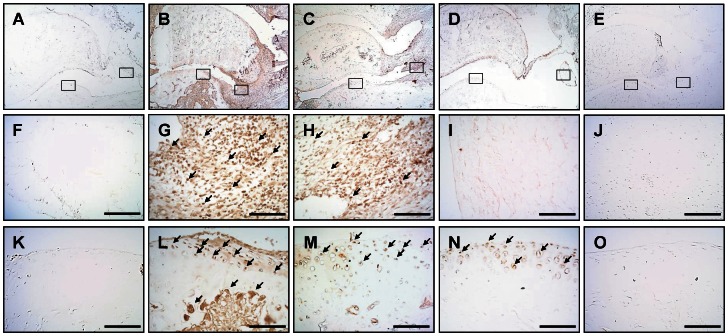
Effect of Ya-s11 on cathepsin K protein expression in the ankle of AIA rats. Cathepsin K protein immunoreactivity is shown in red-brown (arrow) in ankle joint sections from the (A, F, K) naïve, (B, G, L) AIA, (C, H, M) AIA+Ya-s11 (3 mg/kg), and (D, I, N) AIA+Ya-s11 (9 mg/kg) groups. (F)–(J) show cathepsin K immunoreactivity in the synovial tissue of ankle joints outlined in boxes in (A)–(E), respectively. (K)–(O) show cathepsin K immunoreactivity in the articular cartilage outlines in boxes in (A)–(E), respectively. The immunostaining results indicate the upregulation of cathepsin K protein expression in the ankle joint (synovial tissue and cartilage) in AIA rats (B, G, L) and the inhibition of cathepsin K protein expression in synovial tissue and cartilage by treatment with 9 mg/kg but not 3 mg/kg Ya-s11. (E, J, O) A sample from the AIA group incubated without primary antibody for cathepsin K showed no specific staining. Scale bar = 100 µm.

Bone erosion in RA is primarily the result of activated osteoclasts that express enzymes related to bone resorption, such as MMP9, cathepsin K, and TRAP [11], [42]. Figure 6 shows the distribution of cathepsin K immunoreactivity in the ankle joint in the naïve (Fig. 6A), AIA (Fig. 6B), AIA plus Ya-s11 (3 mg/kg) (Fig. 6C), and AIA plus Ya-s11 (9 mg/kg) groups (Fig. 6D). Cathepsin K immunoreactivity appeared to increase in the ankle joint in the AIA group (Fig. 6B). In the AIA plus Ya-s11 (9 mg/kg) group, inhibited AIA-induced upregulation of cathepsin K was observed (Fig. 6D). Higher-magnification images of cathepsin K immunoreactivity in the synovial tissue and cartilage with subchondral bone in the naïve (Fig. 6F, K), AIA (Fig. 6G, L), AIA plus Ya-s11 (3 mg/kg) (Fig. 6H, M), and AIA plus Ya-s11 (9 mg/kg) groups (Fig. 6I, N) are taken from Figures 6A, 6B, 6C, and 6D, respectively. In the AIA group, cathepsin K immunoreactivity was observed in synovial tissue-infiltrating cells (Fig. 6G), cartilage chondrocytes, and subchondral bone marrow (Fig. 6I) of the ankle joint. In the AIA plus Ya-s11 (9 mg/kg) group, cathepsin K immunoreactivity was inhibited in synovial tissue and subchondral bone marrow (Fig. 6I) but not cartilage (Fig. 6N). The AIA plus Ya-s11 (3 mg/kg) group demonstrated reduced cathepsin K immunoreactivity in subchondral bone marrow without inhibition of cathepsin K immunoreactivity in synovial tissue or cartilage (Fig. 6H, M).

**Figure 7:**
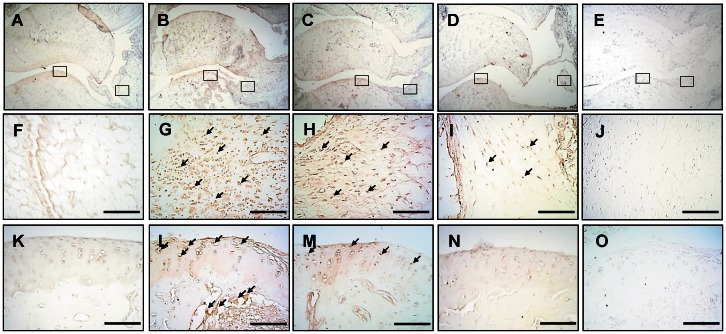
Effect of Ya-s11 on MMP-9 protein expression in the ankle joint of AIA rats. MMP-9 protein immunoreactivity is shown in red-brown (arrow) in ankle joint sections from the (A, F, K) naïve, (B, G, L) AIA, (C, H, M) AIA+Ya−s11 (3 mg/kg), and (D, I, N) AIA+Ya−s11 (9 mg/kg) groups. (F)–(J) show MMP-9 immunoreactivity in the synovial tissue of ankle joints outlined with boxes in (A)–(E), respectively. (K)–(O) show MMP-9 immunoreactivity in the articular cartilage outlined with boxes in (A)–(E), respectively. The immunostaining results indicate the upregulation of MMP-9 protein expression in the ankle joint (synovial tissue and cartilage) in AIA rat (B, G, L) and inhibition of the AIA-induced upregulation of MMP-9 protein expression in synovial tissue and cartilage by treatment with 9 mg/kg but not 3 mg/kg Ya-s11. (E, J, O) A sample from the AIA group incubated without primary antibody for MMP-9 showed no specific staining. Scale bar = 100 µm.

Figure 7 shows the distribution of MMP-9 immunoreactivity in the ankle joint in the naïve (Fig. 7A), AIA (Fig. 7B), AIA plus Ya-s11 (3 mg/kg) (Fig. 7C), and AIA plus Ya-s11 (9 mg/kg) groups (Fig. 7D). In the AIA group, MMP-9 immunoreactivity appeared to increase in the ankle joint (Fig. 7B). Administration of 9 mg/kg but not 3 mg/kg Ya-s11 inhibited AIA-induced MMP-9 expression in the ankle joint (Fig. 7C, D). Higher-magnification images of MMP-9 immunoreactivity in the synovial tissue and cartilage with subchondral bone in the naïve (Fig. 7F, K), AIA (Fig. 7G, L), AIA plus Ya-s11 (3 mg/kg) (Fig. 7H, M), and AIA plus Ya-s11 (9 mg/kg) groups (Fig. 7I, N) are taken from Figures 7A, 7B, 7C, and 7D, respectively. No obvious increase in MMP-9 immunoreactivity in synovial tissue (Fig. 7G), cartilage, or subchondral bone marrow (Fig. 7I) was observed in the AIA group. Compare with the AIA group, the AIA plus Ya-s11 (9 mg/kg) group demonstrated a noticeable decrease in MMP-9 immunoreactivity in synovial tissue, cartilage, and subchondral bone marrow (Fig. 7G, I, L, N). The AIA plus Ya-s11 (3 mg/kg) group demonstrated reduced MMP-9 immunoreactivity in subchondral bone marrow (Fig. 7M) without any apparent inhibition in synovial tissue or cartilage (Fig. 7H, M).

**Figure 8:**
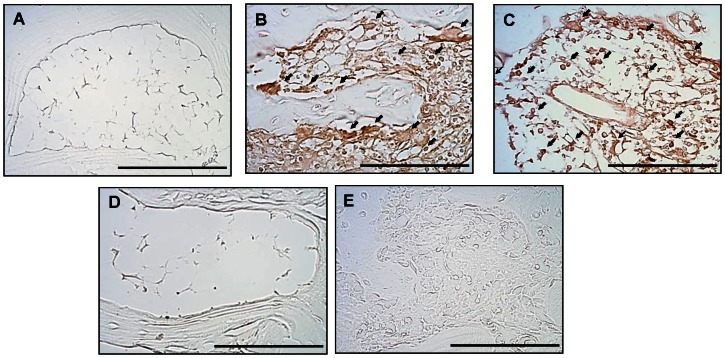
Effect of Ya-s11 on TRAP protein expression in subchondral bone marrow of AIA rats. TRAP protein immunoreactivity is shown in red-brown (arrow) in the bone marrow of ankle joint sections from the (A) naïve, (B) AIA, (C) AIA+Ya−s11 (3 mg/kg), and (D) AIA+Ya−s11 (9 mg/kg) groups. The immunohistochemical results indicate upregulation of TRAP protein expression in the bone marrow of AIA rats (B). Treatment with 9 mg/kg but not 3 mg/kg Ya-s11 appeared to inhibit the AIA-induced upregulation of TRAP protein expression in bone marrow of the ankle joint. (E) A sample from the AIA group reacted without the primary antibody for TRAP showed no specific staining. Scale bar = 100 µm.

**Figure 9:**
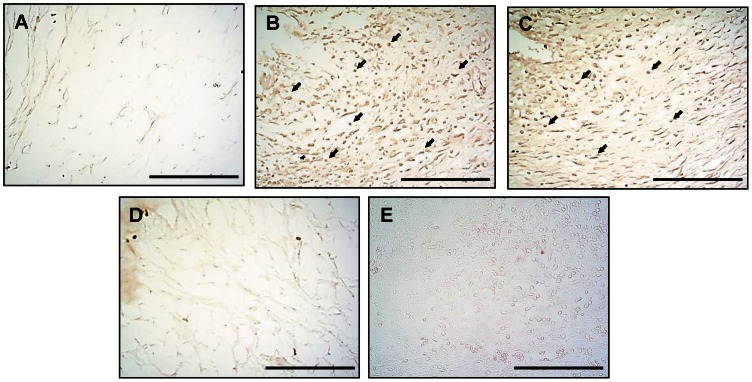
Effect of Ya-s11 on TNF-α protein expression in synovial tissue of AIA rats. TNF-α〈protein immunoreactivity is shown in red-brown (arrow) in the synovial tissue of ankle joint sections from the (A) naïve, (B) AIA, (C) AIA+Ya−s11 (3 mg/kg), and (D) AIA+Ya−s11 (9 mg/kg) groups. The immunohistochemical results indicated upregulation of TNF-α protein expression in synovial tissue in AIA rats (B).Treatment with 9 mg/kg but not 3 mg/kg Ya-s11 appeared to inhibit the AIA-induced upregulation of TNF-α protein expression. (E) A sample from the AIA group reacted without the primary antibody for TRAP showed no specific staining. Scale bar = 100 µm.

Figure 8 shows the distribution of TRAP immunoreactivity in the subchondral bone marrow of the ankle joint in the naïve (Fig. 8A), AIA (Fig. 8B), AIA plus Ya-s11 (3 mg/kg) (Fig. 8C), and AIA plus Ya-s11 (9 mg/kg) groups (Fig. 8D). In the AIA group, TRAP immunoreactivity appeared to increase in bone marrow. Administration of 9 mg/kg but not 3 mg/kg Ya-s11 inhibited AIA-induced TRAP expression in ankle joint bone marrow (Fig. 8C, D). Figure 9 illustrates TNF-α immunoreactivity in synovial tissue from the naïve (Fig. 9A), AIA (Fig. 9B), AIA plus Ya-s11 (3 mg/kg) (Fig. 9C), and AIA plus Ya-s11 (9 mg/kg) groups (Fig. 9D). TRAP immunoreactivity appeared to increase in synovial tissue in the AIA group.

## Discussion

This study employed LPS-induced RAW264.7 murine macrophage cells and AIA as *in vitro* and *in vivo* models, respectively, to assess the anti-inflammatory and anti-arthritic effects of Ya-s11. Marine-derived Ya-s11 significantly down-regulated expression of the proinflammatory proteins iNOS and COX-2 in LPS-induced RAW 264.7 murine macrophage cells. Administration of Ya-s11 also significantly inhibited AIA-induced paw edema and the upregulation of arthritis score in a dose-dependent manner. Histopathological and immunohistochemical examination further demonstrated that AIA-induced histological features in the ankle joint and the osteoclast-related proteins, cathepsin K, MMP-9, TRAP, and TNF-αwere upregulated in ankle joint tissue in the AIA group. Systemic injection of Ya-s11 (9 mg/kg) not only attenuated AIA-induced pathological changes in the ankle joint, but also significantly reduced the osteoclast-related protein expression.

### Effect of Ya-s11 anti-inflammatory activity *in vitro* and *in vivo*

RA is a synovial inflammatory disease characterized by proliferative synovial fibroblasts and infiltrating cells [2]. Previous studies have highlighted the important role played by macrophages in the process of RA [11], [19]. Macrophages mediate synovial inflammation in RA by forming complex cytokine networks with neutrophils, lymphocytes, and synovial fibroblasts and are also critical to osteoclast differentiation [1], [2], [13], [23], [41]. Ya-s11 was able to downregulate iNOS and COX-2 protein expression in LPS-stimulated macrophage RAW 264.7 cells (Fig. 2), a well-established *in vitro* model for assessing the anti-inflammatory activity of compounds. In the *in vivo* study, AIA rats demonstrated a significantly increased number of macrophages in ankle joint synovial tissue as well as AIA-induced increased of neutrophils and lymphocytes with fibroblast proliferation (Fig. 5). Although subcutaneous injection of 3 mg/ml Ya-s11 did not significantly decrease the number of infiltrating cells in synovial tissue, synovial hyperplasia was reduced in this treatment group (Fig. 4). Treatment with 9 mg/ml Ya-s11 inhibited synovial inflammation with reduced cell infiltration in AIA-rats.

### AIA-induced joint destruction with osteoclast-related protein expression

Many studies have clearly illustrated the mediation of joint inflammation, pannus formation, and invasion of infiltrating cells into cartilage and bone by continuous release of osteoclast-related proteins [43]–[45]. TNF-α is a cytokine produced by macrophages that also plays an important role in RA joint destruction and may mediate MMP-9 and cathepsin K expression in RA by upregulating the transcription factor c-Fos/AP-1 [10], [11]. MMP-9 and cathepsin K in turn play important roles in osteoclastogenesis and osteoclastic activity [38], [40], [44] and are expressed by leukocytes, synovial fibroblasts, chondrocytes, and osteoclasts [39], [40], [42], [43], [46], [47]. Our immunohistochemical analyses revealed AIA-induced MMP-9 and cathepsin K expression in synovial tissue, cartilage, and bone marrow (Fig. 6–7). TRAP, which is considered a marker of osteoclasts and plays important roles in osteoclast activity [4], was also observed in bone marrow (Fig. 7). Previous studies have implicated MMP-9, cathepsin K, and TRAP in the bone resorption process in bone marrow [42], [48], and our histopathological assessment of AIA rats also indicated significant bone resorption in the bone marrow (Fig. 4). Hence, the continuous formation of these destructive enzymes in joints affected by RA, with the increase of infiltrating cells, pannus formation, and cartilage and bone erosion and resorption, leads to the development of severe arthritis with joint edema [40], [44]. Accordingly, in the present study, the AIA group demonstrated significant differences in foot and paw edema and clinical evaluation between day 11 and day 28, with edema and erythema of the ankle joint apparent on typical representative macroscopic photographs (Fig. 3).

### Effect of Ya-s11 on AIA-induced joint destruction

We demonstrated that Ya-s11 exerts a therapeutic effect on joint destruction in a rat model of AIA. The therapeutic efficacy of Ya-s11 was not limited to general anti-inflammatory effects, but included substantial inhibition of cartilage and bone destruction compared to AIA rats, as well as inhibition of osteoclast-related protein expression. The AIA plus Ya-s11 (9 mg/kg) group demonstrated inhibition of MMP-9 and cathepsin K expression in the synovial tissue and bone marrow (Fig. 6) as well as inhibited MMP-9 protein expression in articular cartilage. Although cathepsin K expression was not inhibited in chondrocytes, the histological features of the joint display did not display significant morphologic changes in the AIA+Ya−s11 (9 mg/kg) group. TRAP expression in the bone marrow was also inhibited by treatment with Ya-s11 (9 mg/kg). Immunohistochemical analysis further demonstrated an increase in TNF-α in the synovial tissue of the AIA group and its reduction by treatment with Ya-s11 (9 mg/kg). Thus, osteoclast-related protein expression and synovial inflammation were inhibited by Ya-s11, which also demonstrated a dose-dependent effect on foot and paw edema and the clinical evaluation of arthritis and delayed the onset of arthritis. Rats in the Ya-s11 (9 mg/kg) group demonstrated only approximately 10% increase of foot and paw edema compared to baseline, with typical representative macroscopic photographs of the paw implying that edema was not apparent in the photographs, only erythema. In summary, Ya-s11 demonstrates anti-RA activity, with reduced expression of osteoclast activity-related proteins TNF-α, MMP-9, cathepsin K, and TRAP and effective reduction of the clinical features of AIA.

### Ya-s11 as a potential anti-rheumatoid arthritis agent for drug development

The present study demonstrated the efficacy of Ya-s11 as a potential anti-inflammatory compound. We also illustrated its anti-RA activity in AIA model rats, in which Ya-s11 inhibited TNF-α cathepsin K, TRAP, and MMP-9 expression and decreased the major features of RA pathogenesis. Ya-s11 is a cembrane-type natural compound of marine origin originally isolated from the Red Sea [28], [49], which we isolated from the soft coral *Sinularia querciformis*. A cembrane-type compound was first isolated from *Sinularia querciformis* in 1985 [50], and since then 8 types of cembrane-type compound with anti-inflammatory activity have been isolated from this species by Lu et al. [26], [27]. However, Ya-s11 can also be isolated and purified from the same genus of soft coral, *Sinularia flexibilis*, which can be cultured in a culture tank [29], and an increasing number of anti-inflammatory compounds have also been isolated from *Sinularia* sp. [29], [51]–[54]. The main structure of Ya-s11 is a cembranolide analogue, the chemical skeleton of which differs from that of steroids, which may further highlight the potential of Ya-s11 as a useful therapeutic agent for rheumatic diseases and other inflammatory disease.

## Conclusions

In this study, we isolated and purified Ya-s11 from the soft coral *Sinularia querciformis*. *In vitro* study revealed that Ya-s11 significantly inhibited the expression of the proinflammatory proteins iNOS and COX-2 in LPS-challenged murine macrophages cell model. *In vivo* study revealed that Ya-s11 significantly reduced AIA characteristics. Moreover, using histological analysis, we had found that Ya-s11 also improved the histopathologic features of RA. Immunohistochemical analysis showed that Ya-s11 also attenuated protein expression of cathepsin K, MMP-9, TRAP, and TNF-α in ankle tissues of AIA-rats. We concluded that Ya-s11 ameliorated the infiltration of inflammatory cells and bone destruction and downregulated the expression of osteoclast-related proteins in the ankle tissue of AIA rats. Hence, the soft coral-derived compound Ya-s11 may serve as a useful therapeutic agent for the treatment of RA.

## Supporting Information

Figure S1 **Annexin-V/PI double-staining assay.** After treating with different does of Ya-s11, RAW264.7 cells induced by LPS (0.01 µg/ml) were analyzed by fluorescence microscopy by staining with annexin V-FITC and propidium iodide. (A, G) Control group; (B, H) LPS alone group; (C, I) LPS+ Ya-s11 (1 µM); (D, J) LPS+ Ya-s11 (10 µM); (E, K) LPS+ Ya-s11 (25 µM); (F, L) LPS+ Ya-s11 (50 µM). The above observations suggest that different does of Ya-s11 did not induce RAW264.7 cells stimulated with LPS apoptosis or necrosis significantly (red: stained with Annexin V-FITC, green: stained with PI, scale bar = 100 µm).
(TIF)
