# Supplementary figures and images for: A Soft Coral-Derived Compound, 11-epi-Sinulariolide Acetate Suppresses Inflammatory Response and Bone Destruction in Adjuvant-Induced Arthritis
Source: PLoS One. 2013 May 13;8(5):e62926. doi: 10.1371/journal.pone.0062926 (PMC3652811; doi:10.1371/journal.pone.0062926)

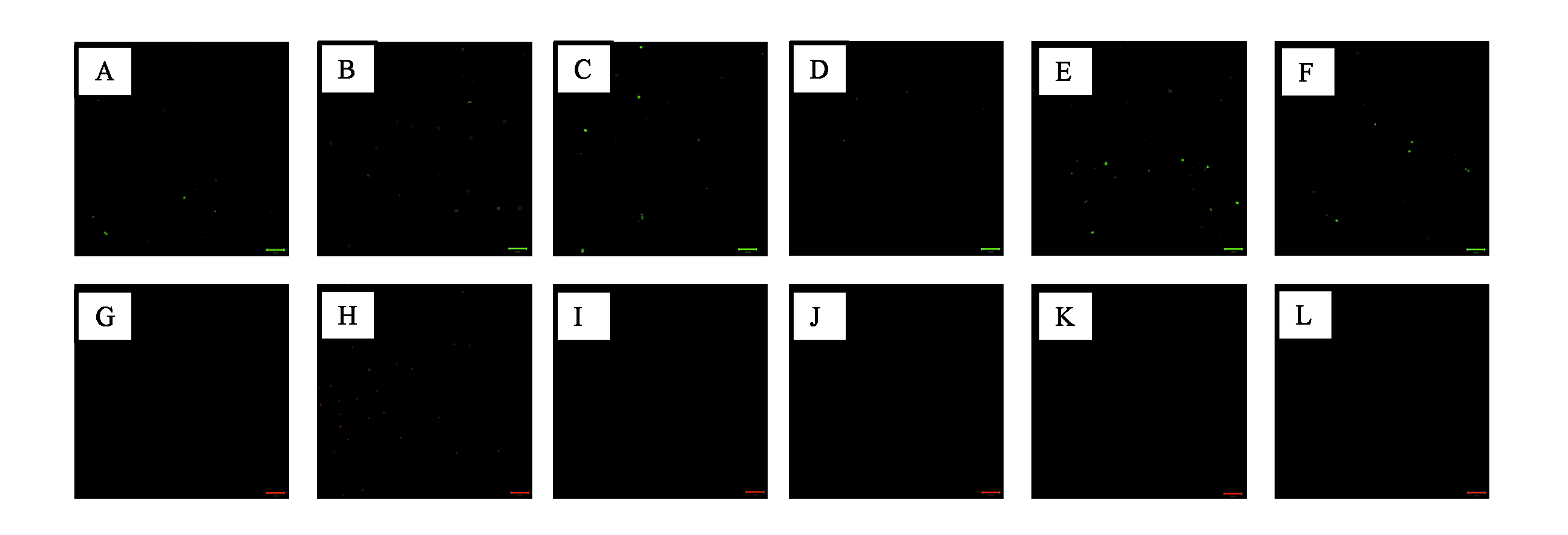

Supplement: Figure S1 — Annexin-V/PI double-staining assay. After treating with different does of Ya-s11, RAW264.7 cells induced by LPS (0.01 µg/ml) were analyzed by fluorescence microscopy by staining with annexin V-FITC and propidium iodide. (A, G) Control group; (B, H) LPS alone group; (C, I) LPS+ Ya-s11 (1 µM); (D, J) LPS+ Ya-s11 (10 µM); (E, K) LPS+ Ya-s11 (25 µM); (F, L) LPS+ Ya-s11 (50 µM). The above observations suggest that different does of Ya-s11 did not induce RAW264.7 cells stimulated with LPS apoptosis or necrosis significantly (red: stained with Annexin V-FITC, green: stained with PI, scale bar = 100 µm). (TIF) [file pone.0062926.s001.tif]
